# Tuberculosis Transmission from Healthcare Workers to Patients and Co-workers: A Systematic Literature Review and Meta-Analysis

**DOI:** 10.1371/journal.pone.0121639

**Published:** 2015-04-02

**Authors:** Monica Sañé Schepisi, Giovanni Sotgiu, Silvia Contini, Vincenzo Puro, Giuseppe Ippolito, Enrico Girardi

**Affiliations:** 1 Department of Epidemiology and Preclinical Research, L. Spallanzani National Institute for Infectious Diseases, Rome, Italy; 2 Epidemiology and Medical Statistics Unit, Department of Biomedical Sciences, University of Sassari, Research, Medical Education and Professional Development Unit, AOU Sassari, Sassari, Italy; 3 Office of the Scientific Director, L. Spallanzani National Institute for Infectious Diseases, Rome, Italy; The Catholic University of the Sacred Heart, ITALY

## Abstract

Healthcare workers (HCWs) are at risk of becoming infected with tuberculosis (TB), and potentially of being infectious themselves when they are ill. To assess the magnitude of healthcare-associated TB (HCA-TB) transmission from HCWs to patients and colleagues, we searched three electronic databases up to February 2014 to select primary studies on HCA-TB incidents in which a HCW was the index case and possibly exposed patients and co-workers were screened.We identified 34 studies out of 2,714 citations. In 29 individual investigations, active TB was diagnosed in 3/6,080 (0.05%) infants, 18/3,167 (0.57%) children, 1/3,600 (0.03%) adult patients and 0/2,407 HCWs. The quantitative analysis of 28 individual reports showed that combined proportions of active TB among exposed individuals were: 0.11% (95% CI 0.04–0.21) for infants, 0.38% (95% CI 0.01–1.60) for children, 0.09% (95% CI 0.02–0.22) for adults and 0.00% (95% CI 0.00–0.38) for HCWs. Combined proportions of individuals who acquired TB infection were: 0.57% (95% CI 7.28E-03 – 2.02) for infants, 0.9% (95% CI 0.40–1.60) for children, 4.32% (95% CI 1.43–8.67) for adults and 2.62% (95% CI 1.05–4.88) for HCWs. The risk of TB transmission from HCWs appears to be lower than that recorded in other settings or in the healthcare setting when the index case is not a HCW. To provide a firm evidence base for the screening strategies, more and better information is needed on the infectivity of the source cases, the actual exposure level of screened contacts, and the environmental characteristics of the healthcare setting.

## Introduction

The transmission of tuberculosis (TB) in healthcare facilities is an important clinical and public health concern. [1, 2].

Since the late 1980s, in the context of rising TB incidence rates in some low- and high-income countries, coupled with the increasing incidence of TB and human immunodeficiency virus (HIV) co-infection, and with the emergence of multidrug-resistant TB (MDR-TB), several major nosocomial TB outbreaks were reported and scientific interest on healthcare-associated transmission of *Mycobacterium tuberculosis* was re-stimulated [3–7]. These outbreaks were attributed to delayed diagnosis of infectious TB patients, unrecognized drug resistance and inadequate infection control measures. On this basis, the major national and international agencies [1, 2, 8] issued recommendations for TB infection control within healthcare facilities, focusing their attention on potential infectious patients as a source of transmission and on protecting other patients and healthcare workers (HCWs).

More recently, episodes of potential transmission of *M*. *tuberculosis* from HCWs to patients have highlighted another risk related to the healthcare-associated transmission of TB (HCA-TB), raising considerable anxiety for the vulnerable groups involved and attracting media attention due to the risk of *M*. *tuberculosis* transmission to numerous contacts. On the other hand, the actual risk of *M*. *tuberculosis* transmission associated to a HCW with TB to the patients he/she serves is still ill-defined [9, 10].

To assess and quantify the magnitude of the risk of transmission of *M*. *tuberculosis* from HCWs with pulmonary TB to patients and co-workers, in order to provide scientific evidence for policy development, we systematically reviewed HCA-TB incidents published to date.

## Methods

We systematically reviewed the medical literature to evaluate the current evidence on the risk of HCA-TB transmission from HCWs. We used the PRISMA (Preferred Reporting Items for Systematic Reviews and Meta-Analyses) statement as a reference document for reporting our findings [11]. The review protocol is available on request from the authors.

### Literature Search

We searched three electronic databases (*i*.*e*. PubMed-MEDLINE, Web of Science, and EMBASE) up to February 2014. Key words used for the electronic search included the following words/lines: “tuberculosis”, “healthcare workers” and “transmission of infectious disease from professional to patient”. Search strategies were adapted for each database. The complete strategy is reported in S1 File.

Reviews or editorials on the topic as well as the lists of the references of the selected articles were analysed to retrieve manuscripts not included in the final output of the bibliographic search. The Outbreak Database (http://www.outbreak-database.com/Home.aspx, last accessed in February 2014), an international database containing standardised data extracted from a large number of outbreak reports published in the medical literature, was checked for additional studies.

A search for the grey literature and media reports, based on the search string: (tuberculosis AND ("healthcare worker" OR "healthcare workers" OR nosocomial OR outbreak)) NOT (bovine OR "non-tuberculous") was attempted using the global electronic outbreak reporting system ProMED-mail (http://www.promedmail.org/), last accessed in February 2014.

### Selection Criteria

In this systematic review, we considered observational studies reporting the results of a contact investigation in a healthcare-associated setting on patients and co-workers exposed to HCWs affected by respiratory active TB.

Reviews, conference contributions, editorials, letters, modelling articles, guidelines/recommendations were excluded.

Selection of the articles was performed by two reviewers (MSS, EG) working independently and following a three-stage procedure: first- titles alone, then abstracts of the selected titles and finally a full-text review of the selected abstracts. Abstracts were discarded only if considered ineligible by both reviewers, otherwise they were retained for the full-text review. Discrepancies were resolved by consensus.

Studies written in any language were considered, but data were extracted only from those published in a language mastered by one of the authors (English, Spanish, Italian, Portuguese or French). When multiple publications from the same population were selected, only those including the most complete report were included. Data extraction procedures are reported in S2 File.

Quality of studies was assessed through the Newcastle-Ottawa quality assessment scale (NOS) for Cohort Studies [12]. The scale consists of nine items that cover three dimensions: (1) patient selection (4 items); (2) comparability of cohorts on the basis of the design or analysis (2 items); and (3) assessment of outcome (3 items). A point is awarded for each item that is satisfied by the study. The total score therefore ranges from zero to nine, with higher scores indicating higher quality. For each study, two checklists were completed, one for each outcome considered (LTBI and active TB).

### Statistical Analysis

The meta-analysis included only those studies reporting results of contact investigation separately for infants (up to 24 months), children (up to 16 years) and adult patients or for health care workers in a single TB incident occurring in a health care facility.

Combined estimates of the proportion of active TB cases and cases who had acquired TB infection resulting from exposure to the HCW, together with their 95% confidence intervals (CIs), were computed collecting all the available data displayed in the selected studies.

A random-effects meta-analysis was performed in order to account for the expected between-study variability for each study, along with a pooled estimate using the softwares for statistical analysis Stata 9.0 (StataCorp. 2005. Stata Statistical Software: Release 9. College Station, TX: StataCorp LP) and StatsDirect 2.8.0.

## Results

**Fig 1:**
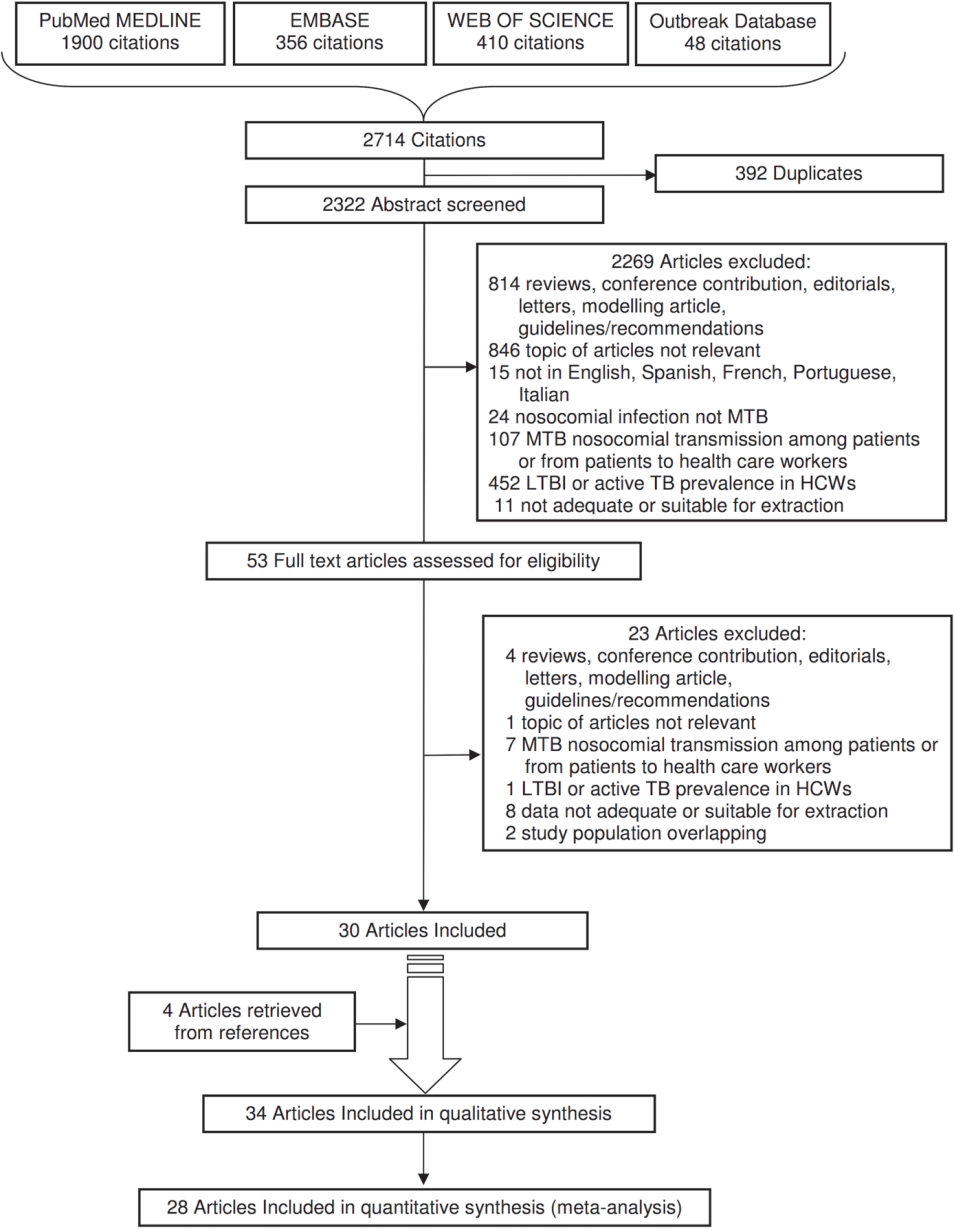
PRISMA 2009 Flow Diagram. Flowchart depicting methods for article inclusion and exclusion. Abbreviations: MTB = Mycobacterium tuberculosis; TB = Tuberculosis; LTBI = Latent Tuberculosis Infection; HCWs = Health Care Workers

Our literature database search yielded 2,714 records and after considering their references and citing articles, we finally included 34 original articles resulting from the selection process described in Fig 1. A complete list of excluded articles with reasons for exclusion is available upon request from the authors.

The 34 articles included in this review reported information on a total of 117 HCA-TB incidents. Twenty-nine articles-thereafter referred to as individual studies- described procedures and findings of contact screening in a single TB incident occurring in a health care facility [13–41]. The remaining five articles—cumulative studies—reported the overall results of retrospective surveys of TB incidents in different settings or cities, identified from national surveillance data [42–46]. Extraction tables are available in S1 Table.

Articles included in the present review were published from 1974 [13, 14] to 2013 [41] and reported data on incidents that occurred in the USA (66, 56.4%) [13–15, 17, 19, 20, 23, 25, 32, 33, 35, 38, 39], France (34, 29.0%) [26, 28, 29, 34, 36, 37, 43, 44, 46], UK (5, 4.2%) [16, 18, 31, 42, 45], Netherlands (6, 5.1%) [22], Canada (2, 1.7%) [21, 24] Ireland (1, 0.9%) [39], Japan (1, 0.9%) [27], Australia (1, 0.9%) [41] and Italy (1, 0.9%) [29].

### Study Quality

Overall, the quality, evaluated by the NOS checklist for the two outcomes “LTBI” and “active TB”, was low (median score four, over a maximum score of nine). The most frequent reasons for low quality scoring were the absence of a non-exposed cohort, the lack of demonstration that the outcome of interest was not present at the start of the study, an absent or too short follow-up for the assessment of the outcome “active TB” and the incomplete follow-up of the cohort when considering exposed adult patients or HCWs. Results of quality assessment of the 34 included studies are reported in S2 Table.

### Description of Included Studies

Nineteen incidents (16.2%) occurred in nurseries/maternity wards [13–15, 17, 22–25, 27–30, 43, 44, 46, 41], 11 in emergency/intensive care/reanimation units [32, 37, 42, 44, 46] (9.4%) and 10 in paediatric units [16, 19, 20, 21, 26, 42–44] (8.5%). Nine incidents (7.7%) occurred in outpatient settings and five involved more than one setting [19, 34, 38, 43, 44].

Characteristics of the 117 index cases are summarised in Table 1.

**Table 1:** Characteristics of 117 Health Care Workers index cases of pulmonary Tuberculosis in 34 included studies.

Characteristics	n.	***(%)***
**Job category**		
nurse/nursing assistant	51	*(43*.*5)*
physician/pediatrician/dentist	22	*(18*.*8)*
Technician	1	*(0*.*9)*
respiratory therapist	1	*(0*.*9)*
Other	5	*(4*.*3)*
data not reported	37	*(31*.*6*)
**Nationality**		
Autochthonous	22	*(18*.*8)*
foreign born	20	*(17*.*1)*
data not reported	75	*(64*.*1)*
**Previous diagnosis of LTBI**		
Known	10	*(8*.*6)*
Unknown	6	*(5*.*1)*
data not reported	101	*(86*.*3)*
**Active TB diagnosis suspected by**		
symptoms (pulmonary, TB, other)	29	*(24*.*8)*
pre-employment screening	4	*(3*.*4)*
intra-employment surveillance	4	*(3*.*4)*
source finding	3	*(2*.*6)*
contact investigation	2	*(1*.*7)*
immigrant screening	1	*(0*.*9)*
data not reported	74	*(63*.*2)*
**Diagnostic interval (days)**		
<30 d	3	*(2*.*6)*
≥30 d	34	*(29*.*0)*
data not reported	80	*(68*.*4)*
**Sputum smear**		
Positive	70	*(59*.*8)*
Negative	12	*(10*.*3)*
data not reported	35	*(29*.*9)*
**Sputum culture**		
Positive	67	*(57*,*2)*
Negative	3	*(2*.*6)*
data not reported	47	*(40*.*2)*
**Drug resistance**		
sensitive to all drugs	40	*(34*.*2)*
any drug resistance	0	
multi-drug resistance	3	*(2*.*6)*
data not reported	74	*(63*.*2)*
**Chest X Ray**		
cavitary lesions present	36	*(30*.*8)*
Absent	44	*(37*.*6)*
data not reported	37	*(31*.*6)*

### Exposed Patients

In 37 out of the 117 incidents, exposed patients were at a high risk of developing active TB once infected with *M*. *tuberculosis* because of young age [13–30, 41–44], oncological-hematological diseases [38], renal diseases [31, 35], haemodialysis [33], HIV infection [34] or other co-morbidities [16, 40].

### Methods and Results of Contact Investigations

In the majority of studies, all individuals who were in the healthcare setting during the period of infectivity of the index case were considered as candidates for screening and no criteria for prioritisation were reported. In seven incidents, priority for screening was defined based on the risk of progression to active TB of exposed individuals [40, 43], while the classic concentric circle approach for contact screening was followed in two incidents [25, 38]. In two incidents [25, 34], the exposed patients were not screened for LTBI, as only surveillance of active TB was performed.

Among individuals identified as candidates for screening, the proportion of those who were actually screened ranged in different studies from 12.8 to 100.0% (median 78.0%) for patients and from 67.3 to 100.0% (median 97.3%) for HCWs.

Tuberculin skin tests were used to detect LTBI, as a single test or combined with other assays (i.e. IGRA not otherwise specified [44], QFT [27], T-SPOT.TB [26]. In two studies, QFT-IT was used as the only test [29] or as the main method in combination with TST [40]. Repeated TST administration to identify the booster phenomenon was used in one study only [33]. (S3 Table)

In 29 individual investigations (S1 Table: Tables A-B), active TB was diagnosed in 3/6,080 (0.05%) infants, 18/3,167 (0.57%) children, 1/3,600 (0.03%) adult patients and 0/2,407 (0.00%) HCWs. Five cumulative studies (S1 Table: Tables C-D) identified active TB in 0/201 (0.00%) infants, 2/2,030 children (0.10%), 1/3,043 adult patients (0.03%) and 3/4,612 HCWs (0.07%). In four reports it was not possible to classify exposed individuals into age groups. Three reports provided data on a total of 2,325 infants and children [20, 21, 44] with no secondary cases, while in an article [18] 15 TB cases were detected among 1,095 exposed children and adults (0.014%).

In one study, the epidemiologic link between the index case and a secondary TB case in an exposed infant was confirmed by IS6110 restriction fragment-length polymorphism (RFLP) [29].

Characteristics of the secondary cases are summarised in Table 2.

**Table 2:** Characteristics of 28 TB cases identified among contacts of Health Care Workers with pulmonary TB.

First Author, year	INDEX CASE		SECONDARY CASES			
	Clinical characteristics	Job category	Site of TB	Type of contact	Age	Concurrent illness or medical procedure at risk
Steiner et al, 1976 [15]	sputum smear neg., culture neg, pulmonary cavitary	nurse’s aide	miliary	patient	3 months	not reported
			miliary	patient	6 months	not reported
Stewart et al, 1976 [16]	sputum smear pos. pulmonary cavitary	physician	pulmonary	patient	2 years	down’s syndrome
			pulmonary	patient	3 years	leukemia
			pleural (disseminated?)	patient	7 years	nephrosclerosis
Smith et al, 1982 [18]	bilateral pulmonary	dentist	palate, cervical adenitis	patient	13 years	tooth extraction
			tooth socket, cervical adenitis, pulmonary	patient	9 years	tooth extraction
			pulmonary	patient	13 years	tooth extraction
			tooth socket, cervical adenitis	patient	6 years	tooth extraction
			cervical adenitis	patient	6 years	tooth extraction
			cervical adenitis	patient	4 years	tooth extraction
			tooth socket, cervical adenitis	patient	32 years	tooth extraction
			cervical adenitis	patient	15 years	tooth extraction
			tooth socket, cervical adenitis	patient	11 years	tooth extraction
			tooth socket, cervical adenitis	patient	8 years	tooth extraction
			tooth socket, cervical adenitis	patient	11 years	tooth extraction
			tooth socket, cervical adenitis	patient	14 years	tooth extraction
			cervical adenitis	patient	11 years	tooth extraction
			cervical adenitis	patient	6 years	tooth extraction
			tooth socket, pulmonary	patient	15 years	tooth extraction
Belfield et al, 1984 [42]	case 1:sputum smear pos. pulmonary cavitary	physician	not reported	patient	'child'	not reported
	case 4: pulmonary	physician	not reported	patient	'child'	not reported
Drobniewski et al, 1995 [31]	sputum smear pos. pulmonary cavitary	staff member	clinical diagnosis, pulmonary	patient	55 yrs	haemodialysis, hepatitis C positive
Noel et al, 2009 [46]	not available	not available	not reported	coworker	not reported	not reported
Migueres et al, 2010 [44]	not available	not available	not reported	patient	'adult'	not reported
			not reported	coworker	'adult'	not reported
			not reported	coworker	'adult'	not reported
Borgia et al, 2011 [29]	sputum smear pos., culture pos. pulmonary	nurse	pulmonary and extrapulmonary (splenic)	patient	4 months	not reported

Long-term follow-up, ranging from 18 months to 4 years, performed by review of TB registers or visiting individuals at risk in eight incidents, did not identify additional active TB cases linked to the index cases [21, 25, 29, 31, 32, 34, 40].

In individual studies, infected cases were 128/5,897 (2.17%) among infants and 236/3,149 (7.49%) among children. Of the 128 infected infants, 118 were from a single study in which only QFT-IT was used to test 1,340 newborns [29]. If the results of this study are excluded, the number of infected infants decreases to 10/4,457 (0.22%). 31/554 (5.6%) adult patients and 30/1,372 (2.18%) HCWs converted to a positive test. (S1 Table: Tables A-B)

In studies for which it was not possible to classify exposed individuals into age groups, 25 infected cases (1.23%) were reported among 2,404 infants and children [21, 22, 44] and 212/1,080 (19.63%) infected individuals were detected among exposed children and adults [18].

In cumulative studies on exposed infants or children (S1 Table: Table C), infected cases were 2/201 (0.01%) and 10/1,613 (0.62%), respectively. Among 2,343 adult patients and 3,515 HCWs (S1 Table: Table D), there were no data on cases converted following exposure to the index cases.

### Meta-analysis

The quantitative analysis included 28 [13–17, 19–41] individual studies for which the proportion of active cases or of infected individuals could be computed separately for infants (up to 24 months), children, adult patients, and/or HCWs.

**Fig 2:**
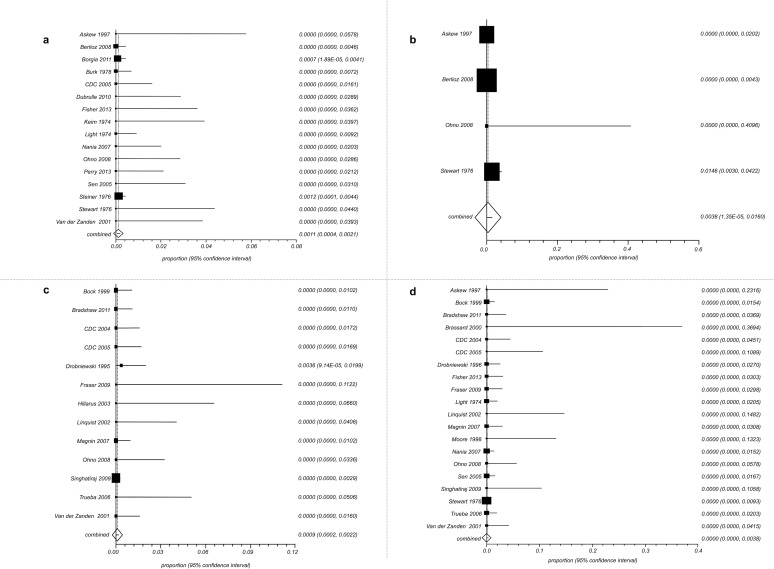
Proportion meta-analysis (random effects). Forest plots for: A. Proportion of active TB cases among infants; B. Proportion of active TB cases among children; C. Proportion of active TB cases among adult patients; D. Proportion of active TB cases among HCWs. (A B C D elements are ordered from top to bottom and left to right).

Combined proportions of active TB cases among exposed individuals were: 0.11% (95% CI 0.04–0.21) for infants (Fig 2A), 0.38% (95% CI 0.01–1.60) for children (Fig 2B), 0.09% (95% CI 0.02–0.22) for adults (Fig 2C) and 0.00% (95% CI C.I 0.00–0.38) for HCWs (Fig 2D).

**Fig 3:**
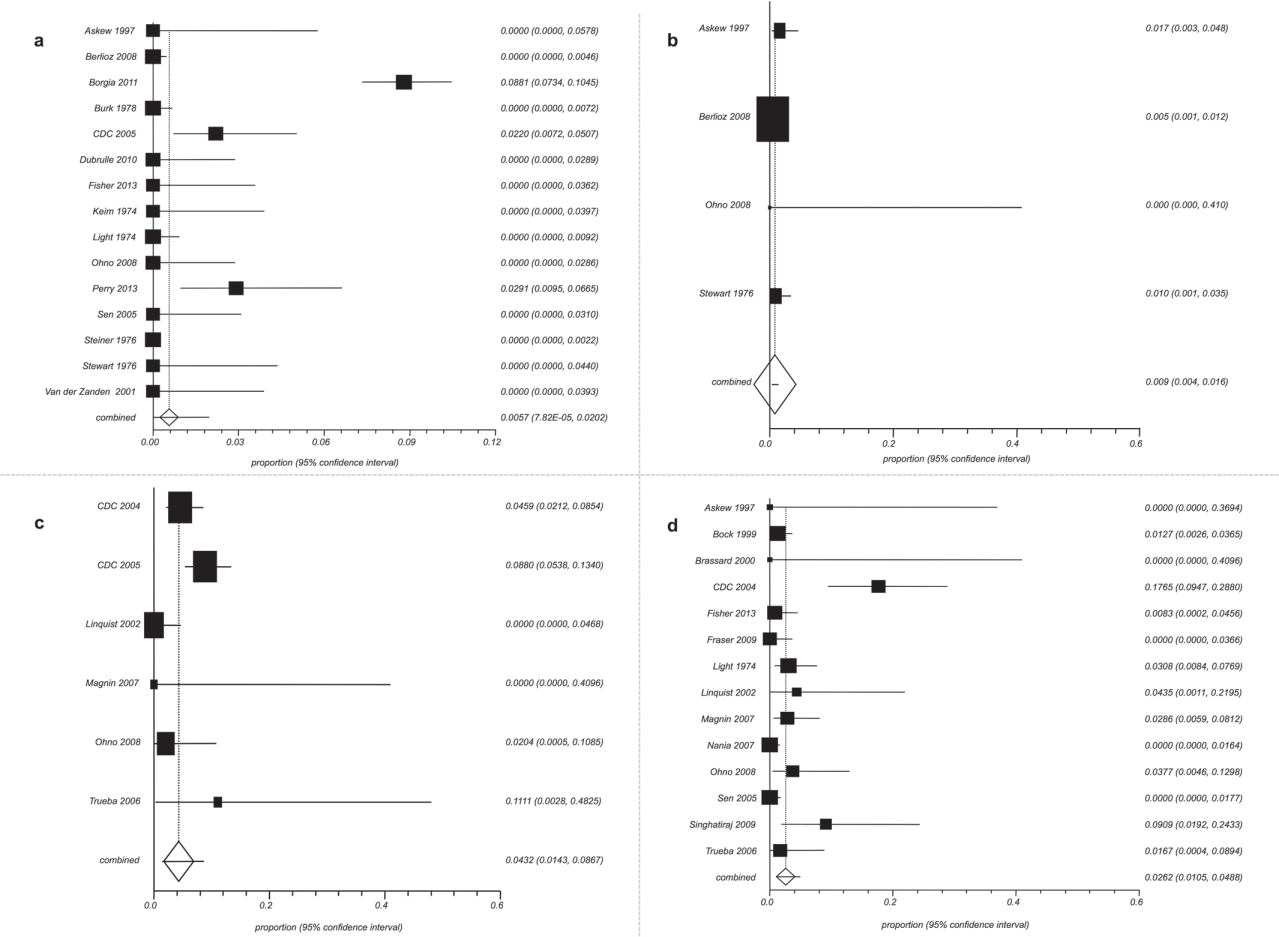
Proportion meta-analysis (random effects). Forest plots for: A. Proportion of cases who acquired TB infection cases among infants; B. Proportion of acquired TB infection among children; C. Proportion of acquired TB infection among adult patients; D. Proportion of acquired TB infection among HCWs. (A B C D elements are ordered from top to bottom and left to right).

Combined proportions of individuals who acquired TB infection were: 0.57 (95% CI 0.00–2.02) for infants (Fig 3A), 0.90% (95% CI 0.40–1.60) for children (Fig 3B), 4.32% (95% CI 1.43–8.67) for adults (Fig 3C) and 2.62% (95% CI 1.05–4.88) for HCWs (Fig 3D). The combined proportion of infants with TB infection was 0.28% (95% CI 0.07–0.65) if the only study (29) in which IGRAs were used to detect LTBI is excluded.

## Discussion

This systematic literature review and meta-analysis has evaluated the magnitude of the risk of transmission of TB from a HCW with pulmonary disease to his/her patients or co-workers in healthcare settings. The evidence identified shows that TB is rarely diagnosed among those exposed to HCWs in a healthcare setting, and that TB infection attributable to this exposure is observed in a limited proportion of exposed individuals. The risk of *M*. *tuberculosis* transmission appears to be clearly lower compared to that recorded in other settings such as schools [47], congregate/community settings [48–54], in the household [55] or even in the healthcare setting when the index case is not a HCW [56–61].

The occurrence of active TB among young children exposed to a potentially infectious person is an important indicator of the risk of transmission of LTBI. In fact, it has been estimated that 50% of the infants aged less than one year and 20–25% of those aged one-two years develop active TB mostly within one year of acquiring the infection [62].

In only two of the 15 incidents included in this meta-analysis in which infants were exposed, secondary active cases were recorded, and the pooled risk of active TB was estimated to be 0.11%. Consistently, we found a very low proportion of exposed infants with TB infection: 0.29% in 13 studies in which the tuberculin skin test was used to screen for TB infection. In a single study [29] involving 1,340 newborns, 8.8% were considered to have acquired TB infection, on the basis of the results of an IGRA test, although only one case of TB was diagnosed among exposed infants at the first screening and no additional cases were recorded after a median follow-up of 18 months (P. Borgia, personal communication). The possibility that false positive IGRA results may have contributed to the high rate of positivity in this study cannot be ruled out. Pooled risks of infection and active disease observed in children aged two to 16 years were also low (0.38% for LTBI and 0.90% for active TB).

The risk of TB transmission to infants and children estimated in this review was clearly lower than that reported in other settings. A recent meta-analysis [55] on the yield of screening of contacts of patients with TB, found a proportion of active TB of 4.7% among children up to five years and 2.9% among children aged six to 14 in high-income countries, and a prevalence of TB infection of 16.3% and 18.4% in the same two age groups. A literature review [47] on school outbreaks involving children aged three to 11 as close contacts reported weighted average TB transmission rates of 39.3% and 69.8% if the index case was an adult or a child, respectively. Very high transmission rates were also recorded in children day-care settings [51–54].

Higher infection transmission rates in HCA settings have also been reported by a systematic review [63] on exposed infants when the index was the mother (1.49%), while lower rates were found when the index case was an infant (0.00%).

Very few active TB cases were diagnosed among exposed adults (two) and co-workers (three). TB infection transmission rates to these individuals, estimated by the conversion to a positive test, (4.32% and 2.62%) were higher than those recorded for children and infants, but lower than those observed in different setting or from a different source. Transmission rates above 20% have been estimated among adults in other congregate settings based on LTBI prevalence [21]. Very high rates of secondary active TB and LTBI transmission have also been reported in nosocomial incidents in which the index case was an HIV infected adult patient [4] or an adult with MDR TB [24]. Information on compliance with recommendations on TB surveillance in HCWs was not provided in many studies. The limited information retrieved however, suggests that this compliance may be suboptimal.

This review has some possible limitations. Despite the comprehensive search, we may have missed relevant studies. Investigation on HCA-TB incidents is a routine public health activity, and therefore does not necessarily result in a scientific publication. In fact, in outbreak databases and global electronic outbreak reporting systems we found some information on incidents for which we could not identify any formal publication. A large proportion of the reviewed incidents involved infants, children, or immunocompromised adults. This may reflect a high propensity to publish incidents involving individuals at increased risk of developing active disease following exposure to TB rather than an increased incidence of TB among HCWs working in these setting, which appears unlikely.

The reliability of figures describing the rates of infection may be somehow limited. In fact there was some heterogeneity in the definitions of TST positivity. Moreover, all included studies considered TST or IGRA positivity in children at the first screening as evidence of LTBI acquired following exposure to the index case. In low incidence countries, this assumption appears plausible in those aged less than two years, while for older children, previous household and community exposures cannot usually be ruled out. For exposed adults, conversion to a positive test was ascribed to exposure to HCW index cases. However, when exposed HCWs were compared to other unexposed personnel in the same or a different hospital [14, 39] or to employees in the same setting during previous years [14] no significant differences in terms of rate of conversion were found.A precautionary approach is usually taken in investigating HCA-TB incidents, resulting in large screenings of potentially exposed patients, which may involve hundreds of persons and require substantial public health resources. On the basis of the low risk of transmission from HCWs evidenced in this review, this approach does not appear to be justified. Effectiveness of alternative strategies, such as initial restriction of screening to those most intensively exposed, extended to those at a lower risk (if transmission is found in the highest-risk group [41]) should be evaluated.

However, to provide a firm evidence base for the screening strategies, more and better information is needed. Results of HCA-TB incident investigations should be made widely available to the scientific and public health community, either through scientific publications or through recording in public health databases [64] and a standardised format for reporting on these incidents should be promoted.

Finally, strategies to promote timely diagnosis of active TB among HCWs may be needed. In this context, the effectiveness of TB symptom reminders to health care staff [65], should be considered.

## Supporting Information

S1 File Search strategy. (DOCX)

S2 File Data extraction. (DOC)

S1 Table Extraction tables. (DOCX)

S2 Table New Castle Ottawa Quality Assessment Scale for cohort studies. (DOC)

S3 Table Tests used to diagnose Latent Tuberculosis Infection and their cut-offs for positivity among patients and co-workers contacts of Health Care Workers with pulmonary tuberculosis. (DOCX)

S4 Table Prisma checklist. (DOC)
