# Supplementary material for: Tuberculosis Transmission from Healthcare Workers to Patients and Co-workers: A Systematic Literature Review and Meta-Analysis
Source: PLoS One. 2015 Apr 2;10(4):e0121639. doi: 10.1371/journal.pone.0121639 (PMC4383623; doi:10.1371/journal.pone.0121639)
Supplement: S1 File — (DOCX) [file pone.0121639.s001.docx]

The following search queries were used:

**Pubmed:**

("Tuberculosis" OR "Mycobacterium tuberculosis" OR "tuberculous"[tiab] OR "tuberculin"[tiab] OR “BCG” OR “Bacillus Calmette-Guerin”) AND ("Infectious Disease Transmission, Professional-to-Patient"[MeSH] OR "nosocomial transmission"[All Fields] OR “nosocomial infections”[ALL FIELDS] OR “health personnel” [MeSH] OR “health care professional” OR “health care worker”); Humans (LIMITS).

**Embase:**

L1 S TUBERCULOSIS+NT/CT

L2 S MYCOBACTERIUM TUBERCULOSIS+NT/CT

L3 S BACILLUS CALMETTE GUERIN/TI,AB

L4 S TUBERCULIN

L5 S L1-L4

L6 S CROSS INFECTION+NT/CT

L7 S NOSOCOMIAL INFECTION#/TI,AB

L8 S HOSPITAL INFECTION+NT/CT

L9 S L5 AND L6-L8

L10 S L5 AND DISEASE TRANSMISSION+NT/CT

L11 S L10 AND PHYSICIAN#(S)PATIENT#

L12 S L9 OR L11

L13 S L12 AND (TUBERCULOSIS/TI OR CALMETTE/TI OR TUBERCULIN/TI)

L14 S L13 NOT ANIMAL#

L15 FOCUS L14 1-

**Web of science:**

tuberculosis and “health care worker*” and transmission
