# Supplementary material for: Tuberculosis Transmission from Healthcare Workers to Patients and Co-workers: A Systematic Literature Review and Meta-Analysis
Source: PLoS One. 2015 Apr 2;10(4):e0121639. doi: 10.1371/journal.pone.0121639 (PMC4383623; doi:10.1371/journal.pone.0121639)
Supplement: S2 File — (DOC) [file pone.0121639.s002.doc]

A predefined spread sheet for data collection was created and the following information was gathered from each included publication: country and setting (in- or out-patient clinic), year of publication and incident period, job category and infectiousness of the index case (*i.e.* identification of a cavitary disease, sputum and/or culture positivity), number of patients and co-workers exposed to the index case, number of patients and co-workers screened, screening method, costs of screening, BCG vaccination status of exposed individuals, number of individuals diagnosed with latent infection (outcome 1) or with active TB (outcome 2) possibly acquired following exposure to the HCW with TB, confirmation of transmission by molecular fingerprinting techniques, and follow-up of exposed individuals for the occurrence of new TB cases. When possible, results of screening of exposed individuals were extracted separately for infants (*i.e.*, up to 24 months), children (*i.e.* up to 16 years) and adults.

The following operational definitions were adopted:

- Index case was defined as a HCW diagnosed with respiratory active TB, regardless of diagnostic criteria, which prompted the contact investigation.

- Latent TB infection (LTBI) possibly acquired following exposure to HCWs in infants or children was defined as a positive result, at baseline or at follow-up testing, of at least one of the following diagnostic tests: Tuberculin Skin Test - TST (including Mantoux test, Tine test and Heaf test), Interferon Gamma Release Assays -IGRAs (including QuantiFERON-TB Gold In Tube (QFT-IT) or T-SPOT.TB). Positivity to TST was defined using the cut-off reported in the original paper.
- LTBI, possibly acquired following exposure to HCWs in adults, was defined as a positive TST or IGRA in an individual with a previously documented negative test performed during the two years preceding exposure or with a baseline negative test performed in the contact investigation. Conversion to a positive TST was defined according to criteria reported in the original paper.
- Secondary case was defined as any person diagnosed with active TB, regardless of diagnostic criteria, during the contact investigation.

Diagnostic interval was defined as the period from the onset of the first symptoms possibly related to pulmonary TB to the date when TB was diagnosed in the HCW.
