# Supplementary material for: Tuberculosis Transmission from Healthcare Workers to Patients and Co-workers: A Systematic Literature Review and Meta-Analysis
Source: PLoS One. 2015 Apr 2;10(4):e0121639. doi: 10.1371/journal.pone.0121639 (PMC4383623; doi:10.1371/journal.pone.0121639)
Supplement: S1 Table — (DOCX) [file pone.0121639.s003.docx]

**A. Included studies reporting individual data on transmission of Tuberculosis from Health Care Workers to infant and children patients**

**and co-workers**

| **First Author, year** | **Setting** | **Index case**  **clinical characteristics (job category)** | **Infant patients** | | | **Children patients** | | | **HCWs** | | | | |
| --- | --- | --- | --- | --- | --- | --- | --- | --- | --- | --- | --- | --- | --- |
|  |  |  | ***% S/E*** | **Active TB/**  **Screened (%)** | **Latent TB infection/**  **Screened (%)** | ***% S/E*** | **Active TB/**  **Screened (%)** | **Latent TB infection/**  **Screened (%)** | ***% S/E*** | **Active TB/**  **Screened (%)** | **Latent TB infection/**  **Screened (%)** | | |
|  |  |  |  |  |  |  |  |  |  |  | ***baseline, prevalent*** | ***documented conversion*** | ***follow up test converted*** |

| Keim, 1974 [13] | hospital nursery | S-, C+, pulmonary cavitary (nurse’s aide) | *nr* | 0/91 | 0/91 | *-* | nr | nr | *-* | nr | nr | nr | nr |
| --- | --- | --- | --- | --- | --- | --- | --- | --- | --- | --- | --- | --- | --- |
| Light, 1974 [14] | hospital nursery | S+, pulmonary cavitary (nurse’s aide) | *90.8* | 0/397 | 0/397 | *-* | nr | nr | *89.9* | 0/178 | nr/130 | 4/130 (3.1) | nr |
| Steiner et al, 1976 [15] | hospital nursery | S-, C-, pulmonary cavitary (nurse’s aide) | *99* | 2/1649 (0.1) | 0/1647 | *-* | nr | nr | *-* | nr | nr | nr | nr |
| Stewart et al, 1976 [16] | hospital pediatric/maternity unit | S+ pulmonary cavitary (physician) | *98.8* | 0/82 | 0/82 | *100* | 3^1^/205 (1.5) | 2^1^/202 (1.0) | *95.4* | 0/394 | nr | nr | nr |
| Burk et al 1978 [17] | hospital nursery | S+ C+ pulmonary (nursery supervisor) | *97.3* | 0/514 | 0/514^2^ | *-* | nr | nr | *-* | nr | nr | nr | nr |
| Smith et al, 1982 [18] | 2 community dental cinics | bilateral pulmonary (dentist) | *-* | nr | nr | *nr* | 15^3^/1095 (1.4) | 212/1080 (19.6) | *-* | nr | nr | nr | nr |
| Askew et al 1997 [19] | private outpatient practice;  day-care center;  3 hospital nurseries | S+ pulmonary cavitary (pediatrician) | *-* | nr | nr | *87.0* | 0/181^4^ | 3/181^4^ (1.7) | *100* | 0/3 | 2/3 (66.7) | nr | nr |
|  |  |  | *96.7* | 0/29 | 0/29 | *-* | nr | nr | *73.3* | 0/11 | 0/11 | nr | 0/8 |
|  |  |  | *58.9* | 0/33 | 0/33 | *-* | nr | nr | *-* | nr | nr | nr | nr |
| Moore et al, 1998 [20] | hospital pediatric unit | S+ C+ pulmonary (pediatrician) | *-* | nr | nr | *68.6* | 0/606^5^ | 14/606^5^ (2.3) | *100* | 0/26 | 1^5^/26 (3.8) | nr | nr |
| Brassard et al, 2000 [21] | private outpatient practice; hospital outpatient clinic | S- C+ pulmonary (pediatrician) | *-* | nr | nr | *48.9* | 0/223^6^ | 1/223 (0.4) | *100* | 0/8 | 1^6^/8 (12.5) | nr | 0/7 |
| Van der Zanden et al,  2001 [22] | hospital obtetrics and ginecology unit | S- pulmonary (student nurse) | *-* | 0/92 | 0/92 | *-* | nr | nr | *-* | 0/87 | 1/87 (1.1) | nr | nr |
| CDC, 2005 [23] | hospital nursery and maternity ward | S+ C+ pulmonary (nurse) | *37* | 0/227 | 5^7^/227 (2.2) | *-* | nr | nr | *100* | 0/32 | 0/7 | nr | nr |
| Sen et al, 2005 [24] | hospital neonatal intensive care unit | S+ C+ pulmonary (physician) | *94.4* | 0/117 | 0/117 | *-* | nr | nr | *100* | 0/219 | 2^8^/206 (1.0) | 0/206 | 0/179 |
| Nania et al, 2007 [25] | hospital neonatal intensive care unit | S- C+ pulmonary (respiratory therapist) | *-* | 0/180 | nr | *-* | nr | nr | *99.2* | 0/241 | 0/223 | nr | 0/223 |
| Berlioz et al 2008 [26] | private outpatient practice; nursery; maternity | S+ C+ pulmonary (pediatrician) | *nr* | 0/803 | 0/803 | *91.3*^9^ | 0/850 | 4^9^/850 (0.5) | *-* | nr | nr | nr | nr |
| Ohno et al, 2008 [27] | hospital newborn nursery and maternity ward | S+ C+ pulmonary (nurse) | *97.7* | 0/127 | 2^10^/127 (1.6) | *100* | 0/7 | 0^10^/7 | *100* | 0/62 | 6^10^/62 (9.7) | 0/nr | 2^10^/53 (3.8) |
| Dubrulle et al, 2010 [28] | hospital neonatology unit | S+ C+ pulmonary cavitary (nursery nurse) | *96.9* | 0/126 | 0/126 | *-* | nr | nr | *-* | nr | nr | nr | nr |
| Borgia et al, 2011[29] | hospital nursery and maternity ward | S+ C+ pulmonary (nurse) | *77.1* | 1/1341 (0.1) | 118^11^/1340 (8.8) | *-* | nr | nr | *-* | nr | nr | nr | nr |
| Perry et al, 2012 [30] | hospital nursery and maternity ward | S+ C+ pulmonary (nurse) | *83.9* | 0/172 | 5/172 (2.9) | *-* | nr | nr | *-* | nr | nr | nr | nr |
| Fisher et al, 2013 [41] | neonatal intensive care unit | S+ C+ pulmonary cavitary (nurse) | *86.2* | 0/100 | 0/100 | *-* | nr | nr | *98.4* | 0/120 | 9/120 (7.5) | 1/120 (0.8) | nr |

**Abbreviations**

%S/E= percentage of individuals screened among those exposed; S=sputum smear; C=sputum culture; nr=not reported; HCWs=Health Care Workers; TST=Tuberculin skin test.

**Notes**

If not otherwise specified in the notes, the diagnostic test used was a Mantoux test.

1: Heaf test (grade 3 or 4). One child who gave a grade 3 Heaf reaction had been given BCG soon after birth.

2: 272 of them tested by Tine method.

3: including 1 adult among active TB cases. The denominator includes adults.

4: includes infants older than 6 months of age. Among 3/181 1 had a documented negative TST, and 2 converted at follow-up test.

5: ranging in age from 1 month to 20 yrs old (median 4.9), exposed only in the outpatient setting. The only TST positive HCW was from a country with a high prevalence of TB and had received BCG; no previous TST result was available.

6: ranging in age from <1 month to 5 yrs old (median 2). The only TST positive child was born in Honduras and had received BCG at birth. The HCW was born in a TB igh prevalence country.

7: including 1 BCG vaccinated.

8: 2 positive HCWs were assumed to be newly positive because there was no documentation of a previous positive result.

9: %S/E available for infants and children together. All T-SPOT.TB were negative.

10: 2 infants aged 0 and 1 year, both BCG-vaccinated were TST positive and QFT negative. Among 127 infants or children<5yrs, 119 were tested by TST only, 2 by QFT only and 6 by both. Among 7 children≥ 5 years 1 was tested by TST alone and 6 by QFT alone. HCWs were screened by QFT.

11: QFT positive and TST negative.

**B. Included studies reporting individual data on transmission of Tuberculosis from Health Care Workers to adult patients and co-workers.**

| **First Author, year** | **Setting** | **Index case**  **clinical characteristics (job category)** | **Adult patients** | | | | | **HCWs** | | | | |
| --- | --- | --- | --- | --- | --- | --- | --- | --- | --- | --- | --- | --- |
|  |  |  | ***% S/E*** | **Active TB/**  **Screened (%)** | **Latent TB infection/**  **Screened (%)** | | | ***% S/E*** | **Active TB/**  **Screened (%)** | **Latent TB infection/**  **Screened (%)** | | |
|  |  |  |  |  | ***baseline, prevalent*** | ***documented conversion*** | ***follow up test converted*** |  |  | ***baseline, prevalent*** | ***documented conversion*** | ***follow up test converted*** |

| Drobniewski et al, 1995 [31] | hospital renal and tranplant centre | S+ pulmonary cavitary (staff member) | *77.9* | 1^1^/277 (0.4 | nr | nr | nr | *73.8* | 0/135 | 1^1^/135 (0.7) | nr | nr |
| --- | --- | --- | --- | --- | --- | --- | --- | --- | --- | --- | --- | --- |
| Bock et al, 1999 [32] | hospital emergency room  hospital outpatient clinic | S+ C+ HIV+ pulmonary (HCW) | *25.6* | 0/175 | 57/175 (32.6) | nr | nr | *nr* | 0/192 | nr/192 | 3/192 (1.6%) | nr |
|  |  | S+ C+ pulmonary (HCW) |  | 0/186 | 40/186 (21.5) | nr | nr |  | 0/45 | nr/45 | 0/45 | nr |
| Van der Zanden et al, 2001 [22] | hospital obtetrics and ginecology unit | S- pulmonary (student nurse) | *-* | 0/228 | 0/228 | nr | nr |  | ^2^ | ^2^ | ^2^ | ^2^ |
| Linquist et al, 2002 [33] | hospital outpatient haemodialysis unit | S+ C+ pulmonary (HCW) | *94.7* | 0/89 | 12/89 (13.5) | nr | 0/77 | *100* | 0/23 | 1/23 (4.3) | 1/23 (4.3) | 0/22 |
| Hillarus et al, 2003 [34] | infectious diseases unit | S+ pulmonary (HCW) | *58.7* | 0/54 | nr | nr | nr | *-* | nr | nr | nr | nr |
| CDC, 2004 [35] | hospital renal dialysis centre | S+ C+ pulmonary (haemodialysis technician) | *72.9* | 0/212 | 29^3^/196 (14.8) | 1/196 (0.5) | 8/175 (4.6) | *84.2* | 0/80 | 13/68 (19.1%) | 6/68 (8.8) | 6/61 (9.8) |
| CDC, 2005 [23] | hospital nursery and maternity ward | S+ C+ pulmonary (nurse) | *24.0* | 0/216 | nr | nr | 19/216 (8.8) |  | ^2^ | ^2^ | ^2^ | ^2^ |
| Trueba et al, 2006 [36] | neurosurgery department | S+ pulmonary cavitary (physician) | *78* | 0/71 | nr/71 | 10^4^/71(14.1) | 1/9 (11.1) | *97.3* | 0/180 | nr/180 | 4^4^/180 (2.2%) | 1/60 (1.7) |
| Magnin et al, 2007 [37] | hospital emergency room | S+ C+ pulmonary cavitary (nurse) | *46* | 0/359 | 20/182 (11.0) | nr | 0/7 | *67.3* | 0/118 | 26/120 (21.7) | nr | 3/105 (2.9) |
| Ohno et al, 2008 [27] | hospital newborn nursery and maternity ward | S+ C+ pulmonary (nurse) | *99.1* | 0/108 | 4/108 (3.7) | nr | 1/49 (2) |  | ^2^ | ^2^ | ^2^ | ^2^ |
| Fraser et al, 2009 [38] | hospital oncology-haematology service (inpatients);  hospital oncology-haematology service (outpatients) | S+ C+ pulmonary (physician) | *61.3* | 0/12 | 0/9 | nr | nr | *81.1* | 0/122 | nr/99 | 0^5^/99 | nr |
|  |  |  |  | 0/19 | 3^5^/10 (30) | nr | nr |  |  |  |  |  |
| Singhatiraj et al, 2009 [39] | hospital outpatient surgery unit | S+ pulmonary cavitary (nurse) | *nr* | 0/1261 | 60/1261 (4.8) | nr | nr | *nr* | 0/33 | nr/33 | 3/33 (9.1) | nr |
| Bradshaw et al, 2011 [40] | hospital | pulmonary MDR (HCW) | *nr* | 0/333 | 30^5^/333 (9.0) | nr | 1^6^/nr | *nr* | 0/98 | 7/98 (7.1) | nr | nr |

**Abbreviations**

%S/E= percentage of individuals screened among those exposed; S=sputum smear; C=sputum culture; nr=not reported; HCWs=Health Care Workers; TST=Tuberculin skin test.

**Notes**

1: clinical diagnosis of TB.: Heaf test. 1/135 HCW had not been vaccinated, but the total of Heaf test positive is not reported.

2: data on exposed HCWs are reported in table A.

3: all 29 patients were considered recently infected, but only 1 previous documented TST result was available.

4: 10 patients and 4/12 positive TST in HCWs were considered LTBI as a) TST>15 mm or b) previous TST negative or increase of more than 10mm induration compared to previous TST, but not clearly specified if a) or b).

5: 3/10 were considered converted by the Authors despite did not have documented baseline TST results. Among 99 HCWs annual TST results were considered as baseline.

6: Quantiferon-In Tube. 2 patients of the 30/333 initially positive then reverted, while 1 initially negative patient converted but finally reverted to negative.

**C. Included studies reporting cumulative data on transmission of Tuberculosis from Health Care Workers to infant and children patients.**

1: all of them already suffering from disease associated with depressed immunity; Heaf test.

2: clinical diagnosis among 220 renal patients.

3: includes infants older than 6 months of age.

4: the 2 HCWs had two distinct molecular patterns.

5: ranging in age from <1 month to 5 yrs old (median 2).

6: visitors.

7: 2 infants aged 0 and 1 year, both BCG-vaccinated were TST positive and QFT negative.

| **First Author, year** | **Setting** | **Index cases considered** | | **Infant patients** | | | **Children patients** | | |
| --- | --- | --- | --- | --- | --- | --- | --- | --- | --- |
|  |  |  |  | ***% S/E*** | **Active TB/**  **Screened (%)** | **Latent TB infection/**  **Screened (%)** | ***% S/E*** | **Active TB/**  **Screened (%)** | **Latent TB infection/**  **Screened (%)** |
|  |  | **n** | **clinical characteristics (job category)** |  |  |  |  |  |  |

| Belfield et al, 1984 [42] | hospital pediatric ward | 2 | case 1:S+ pulmonary cavitary (physician);  case 4:pulmonary (physician); | *-* | na | na | *na* | 2/496 (0.4) | nr |
| --- | --- | --- | --- | --- | --- | --- | --- | --- | --- |
| Carbonne et al, 2005 [43] | 5 hospitals | 2 | case 5: S+ C+ pulmonary (hospital staff)  case 6: S+ C+ pulmonary cavitary (hospital staff) | *12.8* | 0/201 | 2^1^/201 (1) | *12.8* | 0/38 | nr |
| Migueres et al, 2010 [44] | 30 heathcare institutions | 28 | 22 S+, 27 C+ pulmonary, 15 cavitary (10 nurses, 7 physicians, 8 nursing assistant, 3 other HCW) | *-* | na | na | *na* | 0/1496^2^ | 10/1575 (0.6) |

**Abbreviations**

%S/E= percentage of individuals screened among those exposed; S=sputum smear; C=sputum culture; nr=not reported; na=not applicable; HCWs=Health Care Workers; TST=Tuberculin skin test.

**Notes**

1: 2 cases defined as possible LTBI , treated, both BCG vaccinated;

2: cumulative data for infants and children.

**D. Included studies reporting cumulative data on transmission of TB from HCWs to adult patients and co-workers.**

| **First Author, year** | **Setting** | **Index cases considered** | | **Adult patients** | | | | | **HCWs** | | | | |
| --- | --- | --- | --- | --- | --- | --- | --- | --- | --- | --- | --- | --- | --- |
|  |  |  |  | ***% S/E*** | **Active TB/**  **Screened (%)** | **Latent TB infection/**  **Screened (%)** | | | ***% S/E*** | **Active TB/**  **Screened (%)** | **Latent TBinfection/**  **Screened (%)** | | |
|  |  | **n** | **clinical characteristics (job category)** |  |  |  |  |  |  |  |  |  |  |
|  |  |  |  |  |  | ***bas., prev.*** | ***doc. conv.*** | ***follow up test conv.*** |  |  | ***bas., prev.*** | ***doc. conv.*** | ***follow up test conv.*** |
| Carbonne et al, 2005 [43] | 5 hospitals | 3 | case 1: S+ C+ pulmonary cavitary (resident physician)  case 2: S+ C+ pulmonary cavitary (nursing assistant)  case 3: S+ C+ pulmonary (nurse) | *12.8* | 0/297 | nr | nr | nr | *-* | nr | nr | nr | nr |
| Anderson et al, 2007 [45] | 24 incidents in different hospitals | 24 | nr | *-* | 0/nr | nr | nr | 0/nr | *-* | nr | nr | nr | nr |
| Migueres et al, 2010 [44] | 30 heathcare institutions | 28 | 22 S+, 27 C+ pulmonary, 15 cavitary (10 nurses, 7 physicians, 8 nursing assistant, 3 other HCW) | *33.6* | 1/1955 (0.1) | nr | nr | 80/1552^1^ (5.2) | *84.7* | 2/3388 (0.1) | nr | nr | 34/2291 (1.5) |
| Noel et al, 2009 [46] | different services in one health canter | 26 | 11 S+ C+, 2 S+, 5 C+, 3 cavitary; 24 P, 2 EP (19 nurses/ nurse assistant, 5 physicians) | *45.1* | 0/791 | nr | nr | 10/791 (1.3) | *83.5* | 1/1224 (0.1) | nr | nr | 65/1224 (5.3) |

**Abbreviations**

%S/E= percentage of individuals screened among those exposed; S=sputum smear; C=sputum culture; nr=not reported; HCWs=Health Care Workers; TST=Tuberculin skin test; bas.=baseline; prev.=prevalent conv.=converted; P=pulmonary; EP=extrapulmonary.

**Notes**

1: 1497 TST and 55 IGRA.
