# Supplementary material for: Tuberculosis Transmission from Healthcare Workers to Patients and Co-workers: A Systematic Literature Review and Meta-Analysis
Source: PLoS One. 2015 Apr 2;10(4):e0121639. doi: 10.1371/journal.pone.0121639 (PMC4383623; doi:10.1371/journal.pone.0121639)
Supplement: S3 Table — (DOCX) [file pone.0121639.s005.docx]

| **First Author, year** | **Test used** | **screened** | **number and timing of screenings** | | **cut-off for positive value** |
| --- | --- | --- | --- | --- | --- |
| Belfield et al, 1984 [42] | Heaf test | children, adults (HCWs) | 1 | nr | grade 2 |
| Stewart et al, 1976 [16] | Heaf test | infants, children, adults (HCWs) | 1 | nr | grade 2 |
| Drobniewski et al, 1995 [31] | Heaf test | adults (patients and HCWs, if immunocompetent) | 1 | nr | grade 2 |
| Smith et al, 1982 [18] | Heaf test | children and adults (patients) | 1 | nr | grade 2 |
| Burk et al 1978 [17] | Tine test | infants | 1 | at 3m of age | nr |
| Askew et al 1997 [19] | Mantoux test | infants, adults (HCWs) | 2 | 2^nd^ at least at 10 w postexp. | ≥5mm in infants; ≥10mm in adults |
| Berlioz et al 2008 [26] | Mantoux test | infants, children | 2 | 2^nd^ after 12 w postexp. | ≥15mm if BCG vaccinated children or ≥10mm in unvaccinated children |
|  | T-SPOT.TB | children if > 2yrs and intermediate results at follow up TST test: < 5mm 1st and > 5mm 2nd | 1 | nr |  |
| Bock et al, 1999 [32] | Mantoux test | adults (patients and HCWs) | 1 | nr | ≥5mm |
| Bradshaw et al, 2011 [40] | Mantoux test | adults (HCWs) if < 36 yrs and no history of BCG) | 1 | nr | ≥15mm |
|  | Quantiferon | adults (patients and HCWs, if > 36 yrs, BCG vaccinated | 1 to 3 | 6-8 weeks apart,  1 single test in HCWs | ≥0.35 IU/mL |
| Brassard et al, 2000 [21] | Mantoux test | children and adults (HCWs) | 2 | 2^nd^ at 8-10 w postexp. | ≥5mm |
| Burk et al 1978 [17] | Mantoux test | infants | 1 | at 3 m of age | nr |
| CDC, 2004 [35] | Mantoux test | adults (patients and HCWs) | 2 | 2^nd^ after 12 w | ≥5mm |
| CDC, 2005 [23] | Mantoux test | infants, adults (patients and HCWs) | 1 | nr | ≥5mm |
| Carbonne et al, 2005 [43] | Mantoux test | infants, children, adults (patients) | 1 | nr | ≥15mm |
| Dubrulle et al, 2010 [28] | Mantoux test | infants | 2 | 2^nd^ after 12 w | ≥5mm |
| Fisher et al, 2013 [41] | Mantoux test | infants, adults (HCWs) | 1 | infants at 3 m corrected age or 3 m postexp. | ≥10mm or ≥15mm if visible BCG scar |
| Fraser et al, 2009 [38] | Mantoux test | adults (patients and HCWs); baseline test only for family and inpatients | 2 | nr | nr |
| Keim et al, 1974 [13] | Mantoux test | infants | 1 | nr | nr |
| Light et al, 1974 [14] | Mantoux test | infants, adults (HCWs) | 1 | nr | ≥5mm |
| Linquist et al, 2002 [33] | Mantoux test | adults (patients and HCWs) | 3 | 1^st^ and 2^nd^ 1 w apart (booster), then at 12 w | ≥10mm |
| Magnin et al, 2007 [37] | Mantoux test | adults (patients and HCWs) | 2 | 3 m interval | ≥14mm |
| Migueres et al, 2010 [44] | Mantoux test | infants, children, adults (patients and HCWs) | 4 | at 0, 1, 3. 6 m | nr |
|  | IGRA | adults (patients and HCWs) | 4 | at 0, 1, 3. 6 m | nr |
| Moore et al, 1998 [20] | Mantoux test | children, adults (HCWs) | 2 | 2^nd^ at 10 w postexp. | ≥5mm |
| Nania et al, 2007 [25] | Mantoux test | adults (HCWs) | 2 | 2^nd^ at 12 w postexp. | ≥5mm |
| Ohno et al, 2008 [27] | Mantoux test | infants, children | 2 | 2 m interval | ≥5 mm induration or ≥10 erythema (unvacc.)  and ≥15 induration or ≥30 erythema (vacc.) |
|  | Quantiferon | BCG vacc.children, adults (patients and HCWs) | 2 | 2 m inteval | ≥0.35 IU/mL |
| Perry et al, 2013 [30] | Mantoux test | infants | 2 | 3 m interval | ≥10 mm |
| Sen et al, 2005 [24] | Mantoux test | infants, adults (HCWs) | 2 | 2^nd^ in infants at 3 m of age  and in HCW at 3 m | nr |
| Singhatiraj et al, 2009 [39] | Mantoux test | adults (patients and HCWs) | 1 | nr | nr |
| Steiner et al, 1976 [15] | Mantoux test | infants | 1 | nr | ≥10 mm |
| Trueba et al, 2006 [36] | Mantoux test | adults (patients and HCWs) | 2 | 3 m interval | ≥10 mm |
| Borgia et al, 2011 [29] | Quantiferon | infants | 2 | if below 12 weeks of age a 2nd QFT-IT  at the completion of three months of age | ≥0.35 IU/mL |

**Abbreviations**

w=weeks; m=months; nr=not reported; na=not applicable; HCWs=Health Care Workers; QFT-IT=Quantiferon In Tube; TST=Tuberculin skin test; postexp.=postexposure; vacc.=vaccinated; unvacc.=unvaccinated.
