# Supplementary material for: Tuberculosis Transmission from Healthcare Workers to Patients and Co-workers: A Systematic Literature Review and Meta-Analysis
Source: PLoS One. 2015 Apr 2;10(4):e0121639. doi: 10.1371/journal.pone.0121639 (PMC4383623; doi:10.1371/journal.pone.0121639)
Supplement: Supplementary file 6 [file pone.0121639.s004.doc]

		Author, Year	Anderson 2007		Askew 1997		Belfield 1984		Berlioz 2008		Bock 1999		Borgia 2011		Bradshaw 2011	
			LTBI	TB	LTBI	TB	LTBI	TB	LTBI	TB	LTBI	TB	LTBI	TB	LTBI	TB
**1**		**Selection**														
		**1) Representativeness of the exposed cohort**														
	**1a**	**a) truly representative of the average _______________ (describe) in the community**	1	1			1	1								
	**1b**	**b) somewhat representative of the average ______________ in the community**			1	1			1	1	1	1	1	1		
	**1c**	c) selected group of users eg nurses, volunteers													0	0
	**1d**	d) no description of the derivation of the cohort														
**2**		**2) Selection of the non exposed cohort**	na		na		na		na				na		na	
	**2a**	**a) drawn from the same community as the exposed cohort**									1	1				
	**2b**	b) drawn from a different source														
	**2c**	c) no description of the derivation of the non exposed cohort														
**3**		**3) Ascertainment of exposure**														
	**3a**	**a) secure record (eg surgical records)**	1	1	1	1	1	1	1	1	1	1	1	1	1	1
	**3b**	**b) structured interview**														
	**3c**	c) written self report														
	**3d**	d) no description														
**4**		**4) Demonstration that outcome of interest was not present at start of study**		na		na		na		na		na		na		na
	**4a**	**a) yes**														
	**4b**	b) no	0		0		0		0		0		0		0	
**5**		**Comparability**														
		1) Comparability of cohorts on the basis of the design or analysis	na		na		na		na				na		na	
	**5a**	**a) study controls for _____________ (select the most important factor)**									1	1				
	**5b**	**b) study controls for any additional factor (This criteria could be modified to indicate specific control for a second important factor)**														

		Author, Year	Anderson 2007		Askew 1997		Belfield 1984		Berlioz 2008		Bock 1999		Borgia 2011		Bradshaw 2011	
			LTBI	TB	LTBI	TB	LTBI	TB	LTBI	TB	LTBI	TB	LTBI	TB	LTBI	TB
**6**		**1) Assessment of outcome**														
	**6a**	**a) independent blind assessment**	1	1	1	1	1	1	1	1	1	1	1	1	1	1
	**6b**	**b) record linkage**														
	**6c**	c) self report														
	**6d**	d) no description														
**7**		**2) Was follow-up long enough for outcomes to occur**					na	na								
	**7a**	**a) yes (select an adequate follow up period for outcome of interest)**	1		1	1			1	1	1		1		1	
	**7b**	b) no		0		0				0		0		0		0
**8**		**3) Adequacy of follow up of cohorts**		na		na	na	na		na				na		
	**8a**	**a) complete follow up - all subjects accounted for**														
	**8b**	**b) subjects lost to follow up unlikely to introduce bias small number lost - > 70 % (select an adequate %) follow up, or description provided of those lost)**			1				1		1		1			
	**8c**	c) follow up rate < ____% (select an adequate %) and no description of those lost	0												0	
	**8d**	d) no statement										0				0
		**TOTAL SCORE**	**4**	**3**	**5**	**4**	**3**	**3**	**5**	**4**	**7**	**5**	**5**	**3**	**3**	**2**

Abbreviations

LTBI=Latent Tuberculosis Infection; TB=Tuberculosis; na=not available.

		Author, Year	Brassard 2000		Burk 1978		Carbonne 2005		CDC 2004 Nevada		CDC 2005 New York		Drobniewski 1995		Dubrulle 2010	
			LTBI	TB	LTBI	TB	LTBI	TB	LTBI	TB	LTBI	TB	LTBI	TB	LTBI	TB
**1**		**Selection**														
		**1) Representativeness of the exposed cohort**														
	**1a**	**a) truly representative of the average _______________ (describe) in the community**					1	1								
	**1b**	**b) somewhat representative of the average ______________ in the community**	1	1	1	1			1	1	1	1	1	1	1	1
	**1c**	c) selected group of users eg nurses, volunteers														
	**1d**	d) no description of the derivation of the cohort														
**2**		**2) Selection of the non exposed cohort**	na		na		na		na		na		na		na	
	**2a**	**a) drawn from the same community as the exposed cohort**														
	**2b**	b) drawn from a different source														
	**2c**	c) no description of the derivation of the non exposed cohort														
**3**		**3) Ascertainment of exposure**														
	**3a**	**a) secure record (eg surgical records)**	1	1	1	1	1	1	1	1	1	1			1	1
	**3b**	**b) structured interview**											1	1		
	**3c**	c) written self report														
	**3d**	d) no description														
**4**		**4) Demonstration that outcome of interest was not present at start of study**		na		na		na		na		na		na		na
	**4a**	**a) yes**														
	**4b**	b) no	0		0		0		0		0		0		0	
**5**		**Comparability**														
		1) Comparability of cohorts on the basis of the design or analysis	na		na		na		na		na		na		na	
	**5a**	**a) study controls for _____________ (select the most important factor)**														
	**5b**	**b) study controls for any additional factor (This criteria could be modified to indicate specific control for a second important factor)**														

		Author, Year	Brassard 2000		Burk 1978		Carbonne 2005		CDC 2004 Nevada		CDC 2005 New York		Drobniewski 1995		Dubrulle 2010	
			LTBI	TB	LTBI	TB	LTBI	TB	LTBI	TB	LTBI	TB	LTBI	TB	LTBI	TB
**6**		**1) Assessment of outcome**														
	**6a**	**a) independent blind assessment**	1	1	1	1	1	1	1	1	1	1	1	1	1	1
	**6b**	**b) record linkage**														
	**6c**	c) self report														
	**6d**	d) no description														
**7**		**2) Was follow-up long enough for outcomes to occur**					na	na			na					
	**7a**	**a) yes (select an adequate follow up period for outcome of interest)**	1	1	1	1			1			1	1		1	1
	**7b**	b) no		0						0		0		0		0
**8**		**3) Adequacy of follow up of cohorts**						na		na	na	na				
	**8a**	**a) complete follow up - all subjects accounted for**														
	**8b**	**b) subjects lost to follow up unlikely to introduce bias small number lost - > 70 % (select an adequate %) follow up, or description provided of those lost)**	1		1	1			1				1		1	
	**8c**	c) follow up rate < ____% (select an adequate %) and no description of those lost					0									
	**8d**	d) no statement		0										0		0
		**TOTAL SCORE**	**5**	**4**	**5**	**5**	**3**	**3**	**5**	**3**	**3**	**4**	**5**	**3**	**5**	**4**

Abbreviations

LTBI=Latent Tuberculosis Infection; TB=Tuberculosis; na=not available.

	Author, Year	Fisher 2013		Fraser 2009		Hillarus 2003		Keim 1974		Light 1974		Linquist 2002		Magnin 2007	
		**LTBI**	**TB**	**LTBI**	**TB**	**LTBI**	**TB**	**LTBI**	**TB**	**LTBI**	**TB**	**LTBI**	**TB**	**LTBI**	**TB**
	**Selection**														
	**1) Representativeness of the exposed cohort**														
**1a**	**a) truly representative of the average _______________ (describe) in the community**														
**1b**	**b) somewhat representative of the average ______________ in the community**	1	1	1	1	1	1	1	1	1	1	1	1	1	1
**1c**	c) selected group of users eg nurses, volunteers														
**1d**	d) no description of the derivation of the cohort														
	**2) Selection of the non exposed cohort**	na		na		na		na				na		na	
**2a**	**a) drawn from the same community as the exposed cohort**									1	na				
**2b**	b) drawn from a different source														
**2c**	c) no description of the derivation of the non exposed cohort														
	**3) Ascertainment of exposure**														
**3a**	**a) secure record (eg surgical records)**	1	1	1	1	1	1	1	1	1	na	1	1	1	1
**3b**	**b) structured interview**														
**3c**	c) written self report														
**3d**	d) no description														
	**4) Demonstration that outcome of interest was not present at start of study**		na		na		na		na		na		na		na
**4a**	**a) yes**														
**4b**	b) no	0		0		0		0		0		0		0	
	**Comparability**														
	1) Comparability of cohorts on the basis of the design or analysis	na		na		na		na				na		na	
**5a**	**a) study controls for _____________ (select the most important factor)**									1	na				
**5b**	**b) study controls for any additional factor (This criteria could be modified to indicate specific control for a second important factor)**														

	Author, Year	Fisher 2013		Fraser 2009		Hillarus 2003		Keim 1974		Light 1974		Linquist 2002		Magnin 2007	
		LTBI	TB	LTBI	TB	LTBI	TB	LTBI	TB	LTBI	TB	LTBI	TB	LTBI	TB
	**1) Assessment of outcome**														
**6a**	**a) independent blind assessment**	1	1	1	1		1	1	1	1	1	1	1	1	1
**6b**	**b) record linkage**														
**6c**	c) self report														
**6d**	d) no description					0									
	**2) Was follow-up long enough for outcomes to occur**														
**7a**	**a) yes (select an adequate follow up period for outcome of interest)**	1		1		1		1	1	1	1	1		1	
**7b**	b) no		0		0		0		0		0		0		0
	**3) Adequacy of follow up of cohorts**				na				na		na				na
**8a**	**a) complete follow up - all subjects accounted for**														
**8b**	**b) subjects lost to follow up unlikely to introduce bias small number lost - > 70 % (select an adequate %) follow up, or description provided of those lost)**			1						1		1			
**8c**	c) follow up rate < ____% (select an adequate %) and no description of those lost													1	
**8d**	d) no statement	0	0			0	0	0					0		
	**TOTAL SCORE**	**4**	**3**	**5**	**3**	**3**	**3**	**4**	**4**	**7**	**3**	**5**	**3**	**5**	**3**

Abbreviations

LTBI=Latent Tuberculosis Infection; TB=Tuberculosis; na=not available.

		Author, Year	**Migueres 2010**		**Moore 1998**		**Nania 2007**		**Noel 2009**		**Ohno 2008**		**Perry 2013**		**Sen 2005**	
			**LTBI**	**TB**	**LTBI**	**TB**	**LTBI**	**TB**	**LTBI**	**TB**	**LTBI**	**TB**	**LTBI**	**TB**	**LTBI**	**TB**
**1**		**Selection**														
		**1) Representativeness of the exposed cohort**														
	**1a**	**a) truly representative of the average ________________ (describe) in the community**	1	1					1	1						
	**1b**	**b) somewhat representative of the average _______________ in the community**			1	1	1	1			1	1	1	1	1	1
	**1c**	c) selected group of users eg nurses, volunteers														
	**1d**	d) no description of the derivation of the cohort														
**2**		**2) Selection of the non exposed cohort**	na		na		na		na		na		na		na	
	**2a**	**a) drawn from the same community as the exposed cohort**														
	**2b**	b) drawn from a different source														
	**2c**	c) no description of the derivation of the non exposed cohort														
**3**		**3) Ascertainment of exposure**														
	**3a**	**a) secure record (eg surgical records)**	1	1	1	1	1	1	1	1	1	1	1	1	1	1
	**3b**	**b) structured interview**														
	**3c**	c) written self report														
	**3d**	d) no description														
**4**		**4) Demonstration that outcome of interest was not present at start of study**		na		na		na		na		na		na		na
	**4a**	**a) yes**														
	**4b**	b) no	0		0		0		0		0		0		0	
**5**		**Comparability**														
		1) Comparability of cohorts on the basis of the design or analysis	na		na		na		na		na		na		na	
	**5a**	**a) study controls for _____________ (select the most important factor)**														
	**5b**	**b) study controls for any additional factor (This criteria could be modified to indicate specific control for a second important factor)**														

		Author, Year	Migueres 2010		Moore 1998		Nania 2007		Noel 2009		Ohno 2008		Perry 2013		Sen 2005	
			**LTBI**	**TB**	**LTBI**	**TB**	**LTBI**	**TB**	**LTBI**	**TB**	**LTBI**	**TB**	**LTBI**	**TB**	**LTBI**	**TB**
**6**		**1) Assessment of outcome**														
	**6a**	**a) independent blind assessment**	1	1	1	1	1	1	1	1	1	1	1	1	1	1
	**6b**	**b) record linkage**														
	**6c**	c) self report														
	**6d**	d) no description														
**7**		**2) Was follow-up long enough for outcomes to occur**														
	**7a**	**a) yes (select an adequate follow up period for outcome of interest)**	1	1	1	1	1	1	1		1	1	1	1	1	1
	**7b**	b) no		0		0		0		0		0				0
**8**		**3) Adequacy of follow up of cohorts**		na		na		na		na		na				na
	**8a**	**a) complete follow up - all subjects accounted for**					1									
	**8b**	**b) subjects lost to follow up unlikely to introduce bias small number lost - > 70 % (select an adequate %) follow up, or description provided of those lost)**			1						1		1	1	1	
	**8c**	c) follow up rate < ____% (select an adequate %) and no description of those lost	0													
	**8d**	d) no statement							0							
		**TOTAL SCORE**	**4**	**4**	**5**	**4**	**5**	**4**	**4**	**3**	**5**	**4**	**5**	**5**	**5**	**4**

Abbreviations

LTBI=Latent Tuberculosis Infection; TB=Tuberculosis; na=not available.

		Author, Year	Singhatiraj 2009		Smith 1982		Steiner 1976		Stewart 1976		Trueba 2006		Van der Zanden 2001	
			LTBI	TB	LTBI	TB	LTBI	TB	LTBI	TB	LTBI	TB	LTBI	TB
1		**Selection**												
		**1) Representativeness of the exposed cohort**												
	1a	**a) truly representative of the average _______________ (describe) in the community**												
	1b	**b) somewhat representative of the average ______________ in the community**	1	1	1	1	1	1	1	1	1	1	1	1
	1c	c) selected group of users eg nurses, volunteers												
	1d	d) no description of the derivation of the cohort												
2		**2) Selection of the non exposed cohort**			na		na		na		na		na	
	2a	**a) drawn from the same community as the exposed cohort**	1	na										
	2b	b) drawn from a different source												
	2c	c) no description of the derivation of the non exposed cohort												
3		**3) Ascertainment of exposure**												
	3a	**a) secure record (eg surgical records)**	1	1	1	1	1	1	1	1	1	1	1	1
	3b	**b) structured interview**												
	3c	c) written self report												
	3d	d) no description												
4		**4) Demonstration that outcome of interest was not present at start of study**		na		na		na		na		na		na
	4a	**a) yes**												
	4b	b) no	0		0		0		0		0		0	
5		**Comparability**												
		1) Comparability of cohorts on the basis of the design or analysis			na		na		na		na		na	
	5a	**a) study controls for _____________ (select the most important factor)**	1	na										
	5b	**b) study controls for any additional factor (This criteria could be modified to indicate specific control for a second important factor)**												

		Author, Year	Singhatiraj 2009		Smith 1982		Steiner 1976		Stewart 1976		Trueba 2006		Van der Zanden 2001	
			LTBI	TB	LTBI	TB	LTBI	TB	LTBI	TB	LTBI	TB	LTBI	TB
**6**		**1) Assessment of outcome**												
	**6a**	**a) independent blind assessment**	1	1	1	1	1	1	1	1	1	1	1	1
	**6b**	**b) record linkage**												
	**6c**	c) self report												
	**6d**	d) no description												
**7**		**2) Was follow-up long enough for outcomes to occur**	na						na				na	na
	**7a**	**a) yes (select an adequate follow up period for outcome of interest)**			1	1	1		1	1	1			
	**7b**	b) no		0		0		0		0		0		
**8**		**3) Adequacy of follow up of cohorts**	na	na	na	na		na		na				na
	**8a**	**a) complete follow up - all subjects accounted for**												
	**8b**	**b) subjects lost to follow up unlikely to introduce bias small number lost - > 70 % (select an adequate %) follow up, or description provided of those lost)**					1		1					
	**8c**	c) follow up rate < ____% (select an adequate %) and no description of those lost									0			
	**8d**	d) no statement										0	0	
		**TOTAL SCORE**	**5**	**3**	**4**	**4**	**5**	**3**	**5**	**4**	**4**	**3**	**3**	**3**

Abbreviations

LTBI=Latent Tuberculosis Infection; TB=Tuberculosis; na=not available.
